# Von Willebrand Disease and Pregnancy: Management Protocol From Labor to the Postpartum Period

**DOI:** 10.7759/cureus.53465

**Published:** 2024-02-02

**Authors:** Brandon Alejandro Muñoz Vargas, Silvia Juliana Contreras Valero, Rafael Leonardo Aragon Mendoza, Roberto Gallo Roa, Leonardo Jose Enciso Olivera

**Affiliations:** 1 Obstetrics and Gynecology, Hospital Universitario de La Samaritana, Bogotá, D.C., COL; 2 Hematology, Hospital Universitario de La Samaritana, Bogotá, D.C., COL

**Keywords:** von willebrand disease, coagulation disorders, obstetric hemorrhage, blood pathology, high-risk pregnancy

## Abstract

Von Willebrand disease (VWD) is a hemostatic disorder characterized by a quantitative or qualitative deficiency of the Von Willebrand factor (VWF). It affects males and females equally. This pathology has more severe clinical manifestations in females of reproductive age, with a mean age of diagnosis at 19 years. In the pregnant patient, Von Willebrand disease poses an increased risk of complications during labor or the postpartum period, attributed to a higher likelihood of experiencing postpartum hemorrhage and its consequential complications arising from transfusion support and multiorgan injury due to tissue hypoperfusion.

We present the case of a 25-year-old G3P2V2A1 patient with a preexisting diagnosis of Von Willebrand disease prior to gestation. The institutional protocol for managing this condition involved the administration of Von Willebrand factor and factor VIII (FVIII) during vaginal delivery and the postpartum period. This resulted in the effective control of perinatal and postpartum bleeding, with an elevation in Von Willebrand factor levels, thereby avoiding the need for blood transfusions and signs of secondary hypoperfusion.

This case underscores the significance of specialized management for Von Willebrand disease during pregnancy and childbirth, emphasizing adherence to institutional protocols involving specific hemostatic factors. The collaborative efforts of a multidisciplinary team, including hematologists, obstetricians, and other healthcare professionals, are crucial for the comprehensive care of females with this condition during the perinatal period.

## Introduction

Von Willebrand disease (VWD) is a clotting disorder characterized by a quantitative or qualitative deficiency of the Von Willebrand factor (VWF) [1]. Synthesized in endothelial cells and megakaryocytes, VWF plays a key role in primary and secondary hemostasis through its interaction with various platelet adhesion and aggregation receptors. It also participates in secondary hemostasis, acting as a factor VIII (FVIII) transporter to avoid the latter’s activated protein C-mediated breakdown in plasma [2,3].

Although the prevalence of all forms of VWD is estimated at 0.6%-1.3%, the symptomatic form of the disease, which requires specific treatment, affects males and females equally [4], with a mean age of 19 years at the time of diagnosis in females [5]. This condition is classified into three types, the most common being type 1 in 70%-78% of cases, characterized by partial quantitative VWF deficiency. Type 2 is characterized by a qualitative VWF deficiency and is subdivided into subtypes: subtype A, subtype B, subtype M, and subtype N, in which VWF has very low factor VIII affinity. Finally, VWD type 3 is characterized by a complete quantitative VWF deficiency [1]. The disease has an autosomal recessive inheritance pattern in types 2N and 3 and a dominant autosomal pattern in types 1, 2A, 2B, and 2M [3,6].

Diagnosis is usually suspected early in life due to manifestations such as gum bleeding, epistaxis, abnormal uterine bleeding, and, in more severe cases, gastrointestinal bleeding. The diagnosis is confirmed by means of VWF quantification based on antigen levels or VWF and clotting factor VII activity. VWF levels under 30% or under 50% accompanied by bleeding symptoms make the diagnosis, indicating the need to refer to hematology centers for adequate classification [3,7].

As part of the physiological changes during pregnancy, estrogens increase protein synthesis in the liver, leading to an increase in clotting factors VII, VIII, X, XII, VWF, and fibrinogen; shorter thromboplastin time, and lower protein S levels, in order to produce the preventive changes needed to ensure a lower probability of postpartum bleeding [8]. As a result, VWF and factor VIII may increase to normal levels, mainly in type 1 and 2 diseases. During the postpartum period, these levels tend to drop, creating a higher risk of postpartum hemorrhage and the need for blood product transfusion, which may give rise to secondary complications such as respiratory failure, pulmonary edema, acute kidney injury [9], and even maternal death.

There are two treatment options for this disease condition during childbirth and the postpartum period: desmopressin or factor VIII and VWF replacement therapy [10]. However, there are no studies showing the superiority of either of these approaches during pregnancy [11]; therefore, there is an absence of a clear approach, particularly during labor, for preventing postpartum bleeding in these patients. The aim of this article is to present the case of a pregnant 25-year-old female diagnosed with Von Willebrand disease, who had an adequate perinatal outcome, and to describe the institutional protocol used for the management of this condition during childbirth and the postpartum period.

## Case presentation

A 25-year-old female, G3P2V2A1, at 38 weeks of gestation, was admitted to Hospital Universitario de La Samaritana for labor induction. On admission, the patient reported occasional epistaxis that resolved spontaneously, with no other associated symptoms. She had a history of Von Willebrand type 1 diagnosed at 10 years of age, for which she received Von Willebrand factor concentrate on multiple occasions until she reached 19 years of age. In her gynecological history, regular menstrual cycles and no complications during her first pregnancy were reported, although she required factor VIII administration during the postpartum period after delivering a term baby. In 2022, she experienced abnormal uterine bleeding of nonorganic origin, which was managed with VWF and factor VIII, with adequate response to treatment.

A multidisciplinary meeting with the participation of gynecology, maternal-fetal medicine, hematology, and anesthesia was convened in order to implement the childbirth care protocol designed for females with VWD (Table 1) with the aim of lowering the risk of bleeding by means of prophylaxis with Von Willebrand factor and clotting factor VIII concentrate.

**Table 1:** Childbirth care protocol for patients with VWD [1,9,11-16] *Use only in patients with known response to desmopressin VWD, Von Willebrand disease; IM, intramuscular; IV, intravenous

Pregnancy stage	Action	
Prenatal visit: third trimester of pregnancy	Genetic counseling. Order factor VIII and Von Willebrand factor (VWF) levels. Order complete blood count, including platelet count. Monitor fetal well-being: every two weeks after 28 weeks of gestation and every week after week 37. Frequency can increase depending on the associated obstetric risk factors	
38-39 weeks	Admission for labor induction. Multidisciplinary assessment, gynecology, maternal-fetal medicine, hematology, anesthesia, and transfusion service. Reserve blood products. If VWF levels are higher than indicated, no pharmacological prophylaxis will be required. Avoid episiotomy and instrumented delivery. Active childbirth. Oxytocin 10 IU IM at the time of anterior shoulder delivery in vaginal delivery or 30 IU IM in cesarean section. If bleeding, consider using tranexamic acid 1000 mg three times a day	
Pregnancy stage	First-line treatment	Second-line treatment
Active phase of labor	VWD type 1: if VWF is under 50 UI/dL, administer factor VIII/Von Willebrand factor complex at a dose of 50 IU/kg. Repeat the dose after 12 hours if no childbirth	Desmopressin IV 0.3 mcg/kg (maximum dose: 25-30 mcg) every 12-24 hours or intranasal 300 mcg every 12-24 hours*
	VWD type 2: if factor VWF is under 100 IU/dL, apply factor VIII/Von Willebrand complex, at a dose of 50 IU/kg; repeat dose after 12 hours if no childbirth	Desmopressin IV 0.3 mcg/kg (maximum dose: 25-30 mcg) every 12-24 hours or intranasal 300 mcg every 12-24 hours
	VWD type 3: if VWF is under 100 UI/dL, administer factor VIII/Von Willebrand factor complex at a dose of 50 IU/kg; repeat dose after 12 hours if no childbirth has occurred	
Delivery or cesarean section	VWD type 1: if VWF is under 50 IU/dL, administer factor VIII/Von Willebrand factor complex at a dose of 50 IU/kg or desmopressin IV 0.3 mcg/kg (maximum dose: 25-30 mcg) every 12-24 hours or intranasal 300 mcg every 12-24 hours	
	VWD type 2: if VWF is under 100 UI/dL, administer factor VIII/Von Willebrand factor complex at a dose of 50 IU/kg or desmopressin IV 0.3 mcg/kg (maximum dose: 25-30 mcg) or intranasal 300 mcg every 12-24 hours	
	VWD type 3: if VWF is under 100 UI/dL, administer factor VIII/Von Willebrand factor complex at a dose of 50 IU/kg	
Pregnancy stage	Action	Pharmacological treatment
Postpartum day 1	Monitor bleeding and uterine tone in intensive care unit	If VWF is under de 50 IU/dL, administer factor VIII/Von Willebrand factor complex at a dose of 50 IU/kg every 12 hours
Postpartum day 2	Monitor bleeding and uterine tone in intensive care unit	If VWF is under 50 IU/dL, administer factor VIII/Von Willebrand factor complex at a dose of 30 IU/kg every 12 hours
Postpartum day 3	Monitor bleeding and uterine tone in intensive care unit	If VWF is under 50 IU/dL, administer factor VIII/Von Willebrand factor complex at a dose of 20 IU/kg every 12 hours
Postpartum day 4	Monitor bleeding and uterine tone in intensive care unit	If VWF is under 50 IU/dL, administer factor VIII/Von Willebrand complex at a dose of 20 IU/kg every 12 hours
Postpartum days 5-7	Monitoring for bleeding and uterine tone	In case of bleeding and if factor VWF is under 50 IU/dL, administer factor VIII/Von Willebrand factor complex at a dose of 50 IU/kg every 12 hours
Follow-up on day 14	Follow-up appointment, order VWF and clotting factor VIII levels	

Upon admission to the maternal-fetal unit, the laboratory results for 30 weeks showed Von Willebrand factor activity at 3% and clotting factor VIII antibodies at 4% (Table 2). On physical examination, the patient was hemodynamically stable, with concordant fetal well-being determined by fetal biometrics and a biophysical profile of 10/10. On the gynecological examination, the Bishop score was 5. In the step-down obstetric care unit, cervical maturation was indicated using intravaginal dinoprostone 10 mg, followed by intravenous oxytocin infusion titratable until achieving regular uterine activity. During the active phase of labor, the patient received the first dose of factor VIII/Von Willebrand (IMMUNATE®) concentrate at a dose of 50 IU/kg, with a total dose of 4200 IU. Following adequate labor progression, vaginal delivery proceeded uneventfully with the birth of a newborn weighing 2760 g and measuring 47 cm, with an appearance, pulse, grimace, activity, and respiration (APGAR) of 9-9-10. Active birth was allowed with the use of uterotonic drugs, including intramuscular methylergonovine 0.2 mg and sublingual misoprostol 800 mcg. On postpartum day 2, 30 IU/kg was administered every 12 hours with a total daily dose of 2500 IU. On postpartum day 3, 20 IU/kg was administered every 12 hours with a total daily dose of 1680 IU. The postpartum period evolved without complications, and the patient was discharged on day 7 of hospitalization. No additional bleeding episodes occurred during outpatient follow-up.

**Table 2:** Behavior of VWF and factor VIII in pregnancy VWF: Von Willebrand factor

Pregnancy stage	VWF activity	Reference range	Factor VIII	Reference range
30 weeks	3%	40%-200%	4%	50%-150%
38 weeks	3%	40%-200%	4%	50%-150%
Postpartum	41%	40%-200%	No report	50%-150%

## Discussion

Von Willebrand disease is a prevalent condition in females of childbearing age, and during pregnancy, it is associated with a higher risk of postpartum bleeding, exposure to massive transfusion protocols, pulmonary edema, acute renal injury, postpartum bleeding-related maternal mortality, and transfusion-related complications [10].

During the prenatal period, particularly in the third trimester, factor VIII and VWF must be carefully monitored [12] in order to plan for delivery, bearing in mind the pathophysiological changes associated with increased levels of procoagulant factors, which tend to be within the normal range [11], with values as high as 100 IU/dL [12].

Care during delivery should be provided in high-complexity centers where hematology and transfusion services are available, making sure to confirm in advance the availability of medications such as VWF, clotting factor VIII, uterotonics, antifibrinolytics, and blood products.

In order to allow females to have a vaginal delivery, the levels of VWF and factor VIII must be above 50 UI/dL; if females need to undergo cesarean section, Von Willebrand factor levels must be higher than 50 UI/dL, while factor VIII levels must be above 80 UI/dL [1] in cases of type 1 disease. However, in females with type 2 or 3 disease, VWF targets must be higher, aiming at levels above 100 UI/dL [13]. If levels are in normal ranges, no pharmacological prophylaxis is required.

Described treatments for use during the intrapartum phase in this disease include intravenous desmopressin at a dose of 0.3 mcg/kg (maximum dose: 25-30 mcg) or 300 mcg intranasally, reaching a peak 30-90 minutes after its administration, and must be given every 12-24 hours [13]. However, this medication is classified as first-line treatment in Von Willebrand type 1, but no effectiveness has been shown in type 3 disease [9]. Management with VWF and factor VIII at a loading dose of 40-60 IU/kg (in the form of VWF/FVIII 500/200 IU 10/mL) has also been described, and given that our patient had been treated previously with VWF and factor VIII, with an effective response, the same treatment line was continued during pregnancy. These treatments can also be boosted with antifibrinolytics such as tranexamic acid at a dose of 1000 mg three times a day [13] or epsilon-aminocaproic acid at a loading dose of 100-150 mg/kg, followed by infusion of 10-15 mg/kg/hour [1]. However, the preventive use of uterotonics as part of the protocol for hemorrhage control during childbirth has not been described in the literature. Consequently, these types of medications should be used in case of postpartum bleeding due to uterine atony.

It is also important to avoid instrumentation at the time of delivery due to an increased risk of maternal and neonatal bleeding given the genetic inheritance of this disease [14,15]. Prenatal genetic counseling is indicated in these females.

The routine use of oxytocin for active birth can be the same as in the usual childbirth protocol in females with no VWD; however, it needs to be used cautiously when given together with desmopressin because of the increased risk of hyponatremia [9].

Considering the risk of delayed postpartum bleeding in females, close inhospital monitoring is required for 5-7 days, with pharmacological management either with desmopressin or with combined VWF/FVIII medications, as there is greater VWF depletion during this period, increasing the risk of obstetric bleeding in the first 48 hours after delivery [16]. These females require close surveillance in intensive or step-down care units given the risk of hemodynamic instability that may ensue.

According to our protocol, delivery is scheduled at term, between 38 and 39 weeks, in order to avoid unexpected labor, because of the risk of obstetric bleeding. Intrapartum and postpartum management is based on the administration of the combined medication during labor and postpartum, tapering the dose as the risk of postpartum bleeding decreases.

## Conclusions

Pregnant patients with Von Willebrand disease require specialized care from a multidisciplinary team at dedicated centers, including gynecology, maternal-fetal medicine, hematology, intensive care, and transfusion medicine. Key interventions are focused on delivery and postpartum to prevent postpartum hemorrhage. Pharmacological management involves desmopressin, Von Willebrand factor (VWF), and coagulation factor VIII, coupled with uterotonic agents and antifibrinolytics such as tranexamic acid. Optimal treatment depends on predetermining the type of Von Willebrand disease (VWD). Desmopressin is preferred for responsive patients; otherwise, initial management involves VWF and factor VIII. Monitoring in the intensive care unit for at least 48 hours is crucial due to the peak risk of postpartum hemorrhage. A well-defined childbirth management protocol is imperative for these patients.
